# Sympathetic and sensory nerve fiber function in multiple system atrophy and idiopathic Parkinson’s disease

**DOI:** 10.1007/s00415-021-10514-9

**Published:** 2021-03-13

**Authors:** Heidrun H. Krämer, Cora Rebhorn, Christian Geber, Frank Birklein

**Affiliations:** 1 Department of Neurology, Justus Liebig University, Klinikstrasse 33, 35392 Giessen, Germany; 2 DRK Schmerz-Zentrum, Auf der Steig 14-16, Mainz, Germany; 3 Department of Neurology, University Medical Center, Johannes-Gutenberg-University, Mainz, Germany

**Keywords:** PD, MSA, Autonomic failure, Somatosensory function, Small fiber pathology

## Abstract

**Objective:**

To explore small fiber somatosensory and sympathetic function in PD and MSA.

**Methods:**

We recruited 20 PD patients (7 women, median age 65.5 years; IQR 54.75–70.0), 10 MSA patients (4 women; median age 68 years; IQR 66.25–74.0), and 10 healthy subjects (HC; 4 women, median age 68; IQR 59.0–71.0 years). Autonomic testing included forehead cooling, intradermal microdialysis of norepinephrine (NE; 10^–5^; 10^–6^; 10^–7^; and 10^–8^), and orthostatic hypotension (OH); somatosensory testing included quantitative sensory testing (QST) according to the protocol of the German Research Network on Neuropathic Pain (DFNS).

**Results:**

OH occurred more frequently in PD (*p* = 0.018) and MSA (*p* = 0.002) compared to HC. Vasoconstriction responses were stronger in PD compared to MSA during forehead cooling (*p* = 0.044) and microdialysis of physiologically concentrated NE solutions (10^–7^; 10–8; *p* = 0.017). PD and MSA had impaired cold (PD: *p* < 0.01; MSA: *p* < 0.05) and warm detection thresholds (PD and MSA, both *p* < 0.05). The mechanical detection threshold was higher in PD (*p* < 0.01). Conversely, mechanical pain thresholds were decreased in PD and MSA (both *p* < 0.001), indicating mechanical hyperalgesia.

**Conclusion:**

In contrast to MSA, we found evidence of peripheral adrenoreceptor hypersensitivity in PD, probably caused by peripheral sympathetic denervation. Sensory testing revealed peripheral neuropathy and central pain sensitization in PD and MSA. Jointly, our data demonstrate autonomic and somatosensory dysfunction in PD and MSA.

## Introduction

Parkinson’s disease (PD) and multiple system atrophy (MSA) are histologically characterized by phosphorylated α-synuclein (P-α-synuclein) deposits in different brain areas (PD: [4]; MSA: [46]) and peripheral neurons [14].

In PD, autonomic disturbances can precede motor symptoms [41] and progress with disease duration [24]. Meta-iodobenzylguanidine (MIBG) scintigraphy indicates impaired transmitter uptake in peripheral cardiac sympathetic neurons [15, 49], and skin biopsies find a reduction of intraepidermal vegetative fibers with α-synuclein deposits around autonomic structures [14], jointly suggesting disturbances and loss of peripheral autonomic neurons. Other early non-motor phenomena in PD are pain and somatosensory disturbances [45]. Accordingly, a reduction of small sensory nerve fibers in the skin, with preserved large fibers, was detected in PD [13].

MSA presents with progressive autonomic failure, extrapyramidal and pyramidal motor signs, and cerebellar impairment in various combinations [parkinsonian (MSA-P) and cerebellar (MSA-C) subtypes] [16]. Many of the MSA cases become clinically evident by isolated autonomic disturbances before other neurological symptoms occur [28]. It is consensus that the degeneration of central brain stem and midbrain autonomic nuclei [3, 16] leads to autonomic failure, most often of the cardiovascular system. According to the central autonomic failure hypothesis, cardiac MIBG SPECT is normal in most [38, 39] but not all cases [37]. Similar to PD, pain is frequent in MSA, affecting approximately 50–73% of the patients [16, 42] and comprised musculoskeletal, neuropathic, and dystonic components [50]. Deposits of α-synuclein are also present in the skin of MSA-P patients. While peripheral autonomic nerves seem to be spared [12], α-synuclein was found in sensory nerves of the subepidermal plexus [14].

These findings of α-synuclein deposits in the skin suggest that autonomic and sensory symptoms in PD and MSA might be disease related. However, the functional sequels of the peripheral α-synuclein deposits have not been completely evaluated. Therefore, we investigated peripheral small fiber function in MSA and PD patients. Hereby, we could explore whether sympathetic failure can be attributed to peripheral sympathetic nerve dysfunction and whether sensory symptoms are related to peripheral sensory nerve fiber loss or sensitization.

## Materials and methods

We included 20 PD patients (7 women, median age 65.5 years; IQR 54.75–70.0), 10 MSA patients (4 women; median age 68 years; IQR 66.25–74.0), and 10 age- and sex-matched healthy subjects (median age 68; IQR 59.0–71.0 years; 4 women).

Diagnoses of PD were made according to the United Kingdom Parkinson’s disease Society Brain Bank criteria [27]. Complete neurological examination was performed to evaluate the severity of PD and disease staging was achieved using the Hoehn and Yahr scale (H&Y) [26]. The unified Parkinson’s disease scale part III (URPDS-III) was employed to assess motor symptoms [23].

A diagnosis of MSA was made according to diagnostic criteria established at the second consensus conference 2007, which include clinical and neuroimaging features [22]. All patients presented with autonomic failure and motor symptoms including poor levodopa-responsive parkinsonism or cerebellar ataxia. All MSA patients had urinary incontinence as a sign of autonomic failure.

A detailed medical history was obtained from all patients. Medication was not withdrawn for the study. In all HC, the medical history and clinical neurological examination remained uneventful.

All subjects underwent testing in a quiet, temperature- and humidity-controlled laboratory.

### Sympathetic nervous system function

Blood pressure and heart rate (HR) were investigated with a digital sphygmomanometer (WEPA, Hillscheid, Germany) after 15 min resting in a supine position.

#### Forehead cooling

The detailed procedure has been described [35]. In brief, a coated ice pack was placed on the forehead for 20 s, while single-point Laser Doppler measurements (Laser Doppler Imager; Moor Instruments Limited, London, UK) continuously assessed acral skin blood flow at the tip of the index finger (sampling frequency 20 Hz, time constant 0.1 s, and distance to skin surface 50 cm). The mean flux value of 30 s before cooling was used as a baseline. Acral vasoconstriction was then analyzed for another 20 s during forehead cooling. The relative change in perfusion units was normalized to the baseline (flux value at baseline = 100).

#### Orthostatic hypotension (OH)

After lying in a supine position for 15 min, blood pressure and heart rate were measured using a digital sphygmomanometer. Then, blood pressure and heart rate were recorded immediately after getting up and again after 1, 2, 3, and 5 min. The test was considered pathologic if the SBP decreased ≥ 20 mmHg or the DBP decreased ≥ 10 mmHg within 3 min after standing up [17].

#### Norepinephrine (NE) microdialysis

The exact procedure has been described [30]. In brief, four microdialysis fibers (DermalDialysis, Erlangen, Germany) were inserted intradermally at a distance of 3 cm to a length of 1.5 cm by a 25-gauge cannula in the right ventral thigh 20 cm above knee level. Each fiber was perfused with a different concentration of norepinephrine (NE; 10^–5^; 10^–6^; 10^–7^; 10^–8^; flow rate: 4 µl/min) by a microdialysis pump (Pump 22; Harvard Apparatus). The high NE concentrations (10^–5^ and 10^–6^) were analyzed together, as were the physiological NE concentrations (10^–7^ and 10^–8^). After 60 min, when insertion-related vasodilation had subsided [1], NE perfusion of the microdialysis membranes was started.

Superficial blood flow at the microdialysis site was quantified using a laser Doppler imager (LDI, Moor, London, U.K.). LDI scans (256 × 256 pixels, scan resolution 4 pixels/s, distance to skin surface: 50 cm; scanned area: 144 cm^2^) were recorded at baseline (three baseline pictures in total) and at intervals of 5 min after the beginning of the NE perfusion. The mean baseline flux value in perfusion units was calculated as the mean from the three acquired baseline pictures. The intensity of the vasoconstriction was analyzed offline (MLDI 3.0; Moor, London, U.K.). Vasoconstriction expressed in flux values was normalized to the baseline (flux value at baseline = 100).

### Quantitative sensory testing (QST)

QST was performed at the more affected hand in PD and MSA and on the dominant hand in healthy controls. Testing was conducted according to the standardized test battery for QST [43]. A brief description is given below (for a more detailed description see [43, 44]).

#### Thermal testing

Thermal detection thresholds (averaged across three repeated trials) for the perception of cool (CDT) and warm (WDT) were recorded using a TSA 2001-II (MEDOC, Ramat Yishai, Israel) with a thermode of Peltier elements (contact area 30 × 30 mm; 32 °C baseline temperature; ramp rate 1 °C/s; method of limits). The number of paradoxical heat sensations (PHS; i.e., reports of hot or burning sensations to innocuous cold stimuli) was determined during the thermal sensory limen (TSL; the difference limen for alternating warm and cold stimuli) procedure. Thermal pain thresholds [cold pain threshold (CPT); heat pain threshold (HPT)] were tested with the same device and in the same fashion. The mean threshold temperature of the three consecutive measurements was calculated.

#### Mechanical thresholds

The mechanical detection thresholds (MDT) were investigated using a standardized set of modified von Frey hairs (Optihair2-Set, Marstock Nervtest, Germany; forces between 0.25 and 512 mN; 0.5 mm in diameter). Mechanical pain thresholds (MPT) were obtained employing suprathreshold mechanical pain sensation (seven forces 8, 16, 32, 64, 128, 256, and 512 mN; flat contact area, 0.25 mm in diameter; PinPrick; MRC Systems GmbH, Germany). An adaptive method of limits by series of alternating ascending and descending stimuli intensities yielding five just suprathreshold and five just subthreshold estimates was used. The final threshold was the geometric mean of the 10 estimates.

A stimulus–response function for mechanical pain sensitivity (MPS) was determined using the same pinprick stimuli. In addition, pain in response to light touch [dynamic mechanical allodynia (DMA)] was tested by gentle/light stroking with a cotton wisp (3 mN), a cotton wool tip fixed to an elastic strip (100 mN), and a brush (200–400 mN). Each of the seven pinpricks and the three types of light stroking were applied five times in a balanced sequence. The MPS was calculated as the geometric mean of all pain ratings for pinprick stimuli, and allodynia was quantified as the geometric mean of all pain ratings after light touch stimuli.

The vibration detection threshold (VDT) was investigated at the processus styloideus ulnae on the upper extremity with a Rydel–Seiffert tuning fork (64 Hz, 8/8 scale). The final vibration detection threshold was the arithmetic mean of three consecutive measurements.

The wind-up ratio (WUR) assessed pain summation to repetitive pinprick stimuli (i.e., pain after 10 stimuli repeated at 1 Hz vs. pain to a single pinprick stimulus at a standard force of 256 mN).

The pressure pain threshold (PPT) at the thenar eminence (upper extremity) was measured using a hand-held blunt pressure gauge device (1 cm^2^ contact area) with an upper load limit of 20 kg (FDN200, Wagner Instruments, USA; ramp rate: 50 kPa/s).

### Statistics

Data were analyzed using the SPSS Statistics (IBM, Version 27.0 for Windows) software package. For the analysis of skin perfusion, repeated-measures analysis of variances (rm-ANOVA) was applied using the factors ‘disease’ (PD, MSA, and HC), ‘concentration’ (NE concentration: 10^–5^ and 10^–6^; 10^–7^ and 10^–8^), and ‘time’. Furthermore, rm-ANOVA was calculated for the forehead cooling analysis (factor ‘disease’: PD, MSA, and HC). The Greenhouse–Geisser correction was applied when the assumption of sphericity was violated. One-way ANOVA was calculated to detect differences in resting cardiovascular parameters between the groups (factor: disease: PD, MSA, and HC). Chi-squared tests were employed to evaluate differences in the occurrence of OH between the groups. Kolmogorov–Smirnov tests of normality were run for all data sets, and parametric or nonparametric statistics were used accordingly, as described in the experiment-specific results.

QST data were z-transformed into a standard normal distribution (zero mean, unit variance) for each single parameter to allow a comparison of QST parameters independent of their physical units using the following expression (except DMA and PHS): Z = (valuepatient − meancontrols)/SDcontrols.

Z-scores below zero indicate a loss of function; z-scores above zero indicate a gain of function. Thus, elevations of thresholds (CDT, WDT, TSL, HPT, CPT, PPT, MPT, MDT, and VDT) result in negative z-scores, whereas increased ratings (MPS and WUR) result in positive z-scores. One-way ANOVAs with LSD post hoc tests were calculated to identify differences between the three investigated groups (factor: disease: PD, MSA, and HC).

Additionally, the three groups (PD, MSA, and HC) were compared to the normative data set of the German network on neuropathic pain (DFNS), as described by Magerl and coworkers [32]. In brief, comparison between records of test group data (PD, MSA, and HC) and a matched control group created as a fictitious subpopulation of reference group data of equal number is performed by *t* test statistic. The distribution of z-values of the control group is always given as mean = 0 and standard deviation (SD) = 1 (see, e.g., [19]).

All values are given as medians and interquartile range (IQR) in the case of a non-normal distribution and as means ± standard error in the case of a normal distribution. Values were considered significant if *p* < 0.05.

## Results

We included 20 patients suffering from idiopathic Parkinson’s disease (H&Y 1: *n* = 2; H&Y 2: *n* = 14; H&Y 3: *n* = 4; URPDS-III: median 10.0 (IQR 7.0–25.0); disease duration: median 4 years (IQR 2–7 years)]. The median of the daily L-dopa equivalent dose was 231 mg (IQR 100–578.125) [51]. 10 MSA patients all diagnosed with ‘probable MSA’ [MSA-P: *n* = 5; MSA-C: *n* = 5; disease duration: median 4 years (IQR 1–6 years)], and 10 age- and sex-matched healthy subjects also participated in the study. For details, see Table 1.

**Table 1:** Clinical and biographical data of the PD and MSA patients as well as the healthy controls

	PD patients (*n* = 20)	MSA patients (*n* = 10)	Healthy participants (*n* = 10)
Age (median)	65.5 years [IQR 54.75–70.0]	68.0 years [IQR 66.25–74.0]	68.0 years [IQR 59.0–71]
Sex (w/m)	7/13	4/6	4/6
Disease stage	H & Y 1: *n* = 2	Probable MSA: *n* = 10	n. a
	H & Y 2: *n* = 14	MSA-C: *n* = 5	
	H & Y 3: *n* = 4	MSA-P: *n* = 5	
Disease duration (median)	4 years [IQR 2–7]	4 years [IQR 1–6]	n. a
L-dopa equivalent daily dose	231 mg [IQR 100–578.125]	n. a	n. a
URPDS-III (median)	10.0 [IQR 7.0–25.0]	n. a	n. a

*PD* Parkinson’s disease, *MSA* multi system atrophy, *IQR* interquartile range, *m* men, *w* women, *H & Y* Hoehn & Yahr scale, *MSA-C* cerebellar MSA-C subtype, parkinsonian MSA subtype, *n. a*.: not applicable, *URPDS* unified Parkinson’s disease scale part III

### Sympathetic nervous system function

At rest, DBP (PD 85 ± 2.4 mmHg; MSA 82 ± 2.4 mmHg; controls 75 ± 2.3 mmHg), SBP (PD 137 ± 4.6 mmHg; MSA 150 ± 7.2 mmHg; controls 131 ± 4.3 mmHg), and HR (PD 66 ± 3.5 beats/minute; MSA 66 ± 2.7 beats/minute; controls 70 ± 2.6 beats/minute) were not different between groups (one-way ANOVA, ns).

#### Forehead cooling

**Fig. 1:**
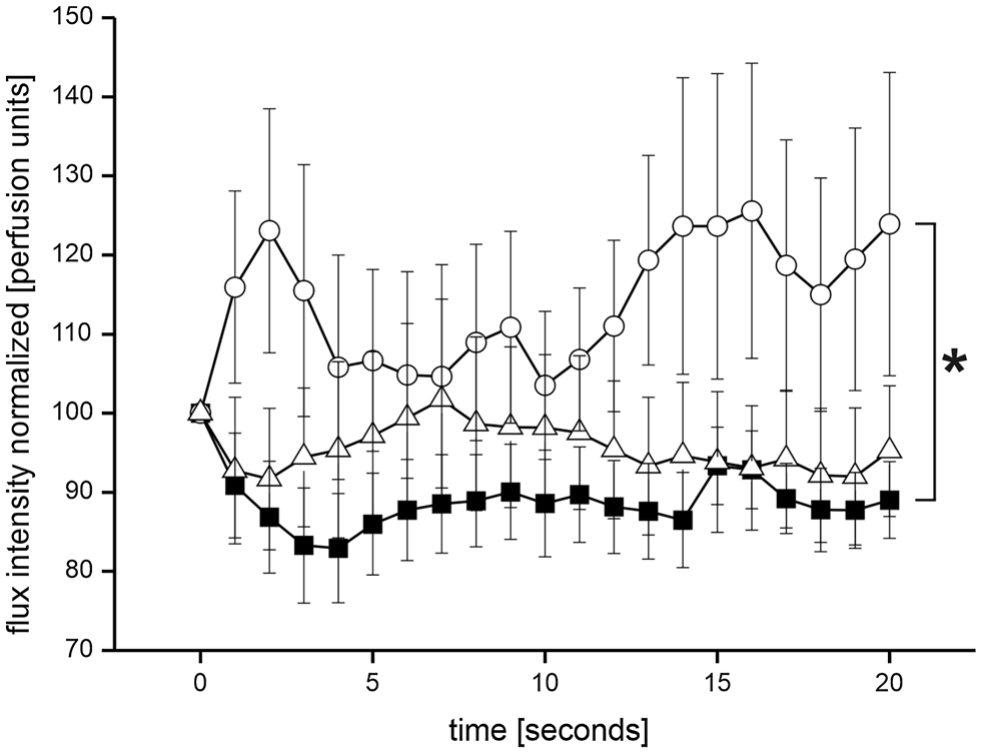
Shows the cold-induced acral vasoconstriction over time during forehead cooling in healthy controls (open triangles), PD patients (filled squares), and MSA patients (open circles). The cold-induced vasoconstriction response is stronger in PD compared to MSA (*F* = 4.106; *p* = 0.044; rm-ANOVA). *p < 0.05

ANOVA analysis of all three groups together did not reveal differences in cold-induced vasoconstriction responses. However, if PD and MSA were directly compared in a post hoc ANOVA analysis, PD responded with vasoconstriction, but MSA patients had an increase of blood flow indicating sympathetic vasoconstriction failure (*F* = 4.106; *p* = 0.044; rm-ANOVA). No difference between healthy controls and MSA or PD could be detected (rm-ANOVA, ns). For details, see Fig. 1.

#### Orthostatic hypotension

OH was present in 11 of the 20 PD and in 8 of the 10 MSA patients. Formally, 1 of 10 HC also had OH. The presence of OH differed between the three groups (Chi-square test 10.2; *p* = 0.006). OH occurred more frequently in PD (Chi-square test 5.625; *p* = 0.018) and MSA (Chi-square test 9.899; *p* = 0.002) compared to HC. The occurrence of OH did not differ between PD and MSA patients (Chi-squared test; ns).

#### Norepinephrine microdialysis

Baseline skin perfusion at the thigh did not differ between the three different groups (PD 143.40 ± 8.29 PU; MSA 127.79 ± 9.37 PU; HC 125.87 ± 5.63 PU; one-way ANOVA, ns).

**Fig. 2:**
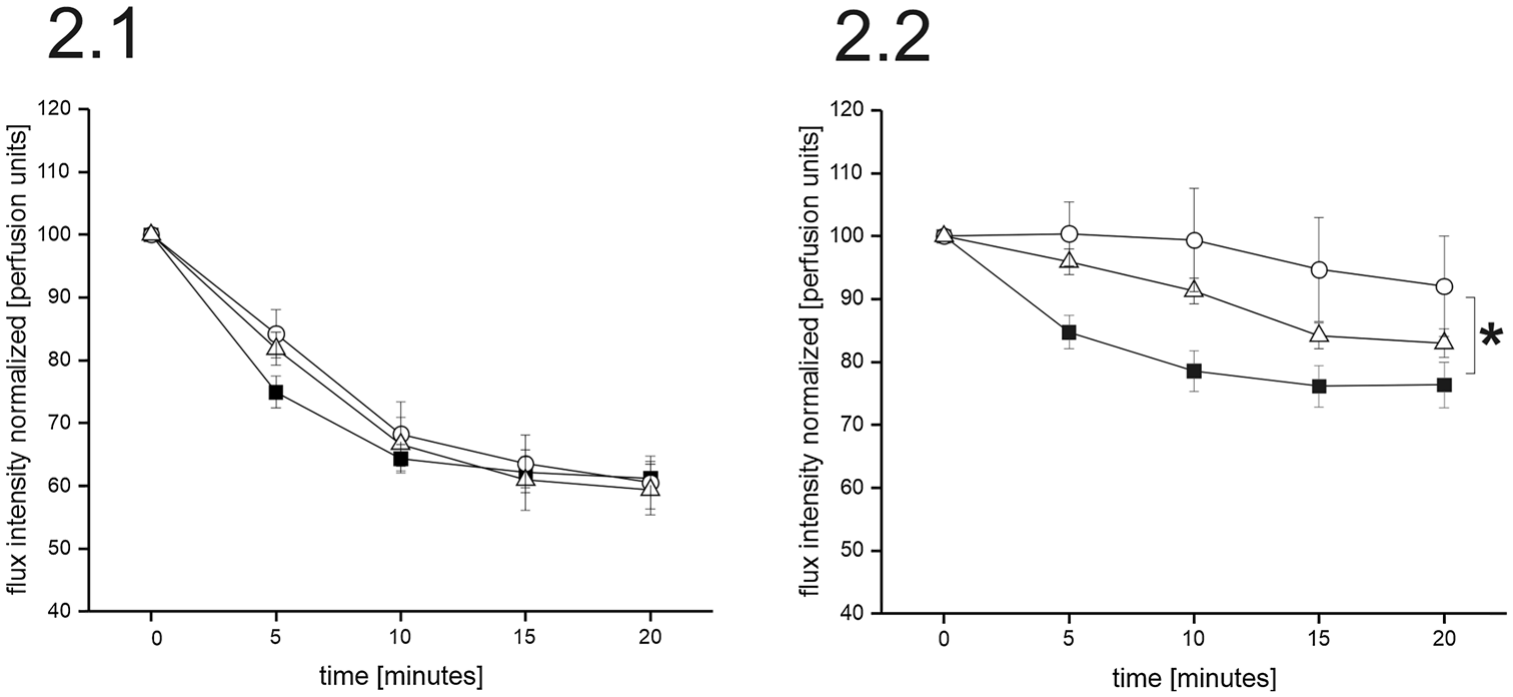
Represents the time course analysis of the degree of vasoconstriction induced by the applied NE concentrations [Fig. **2.1** high NE concentrations (10^–5^ and 10^–6^); Fig. **2.2** physiological NE concentrations (10^–7^ and 10^–8^)] in healthy controls (open triangles), PD patients (filled squares), and MSA patients (open circles). The degree of vasoconstriction is represented in flux intensity normalized to baseline in arbitrary perfusion units. **2.1** High NE concentrations override differences in vasoconstrictive capacities (rm-ANOVA, ns). **2.2** NE-induced vasoconstriction significantly differed between the three groups (*F* = 4.308; *p* = 0.017). Post hoc tests revealed that vasoconstriction in PD is stronger than in MSA (*p* = 0.006; LSD), indicating supersensitivity of NE receptors possibly due to denervation. *p < 0.05

As expected, norepinephrine (NE) leads to dose-dependent vasoconstriction (*F* = 37.595; *p* < 0.001; rm-ANOVA) in all three groups. While pharmacologically high concentrations of NE (10^–5^ and 10^–6^) led to identical vasoconstriction in all groups, vasoconstriction induced by physiological NE concentrations (10^–7^ and 10^–8^) significantly differed between the three groups (*F* = 4.308; *p* = 0.017): vasoconstriction in PD was more effective than in MSA which showed no vasoconstriction at all (*p* = 0.006; LSD post hoc test). HC results were in the middle between both patient groups. This result indicates different arteriolar NE-receptor sensitivity between patient groups. For details, see Fig. 2.

### Somatosensory profiles (QST)

None of the PD or MSA patients reported sensory symptoms or any clinical signs of small fiber neuropathies, such as neuropathic pain [11].

#### Comparison of QST parameters to the DFNS normative data set

**Fig. 3:**
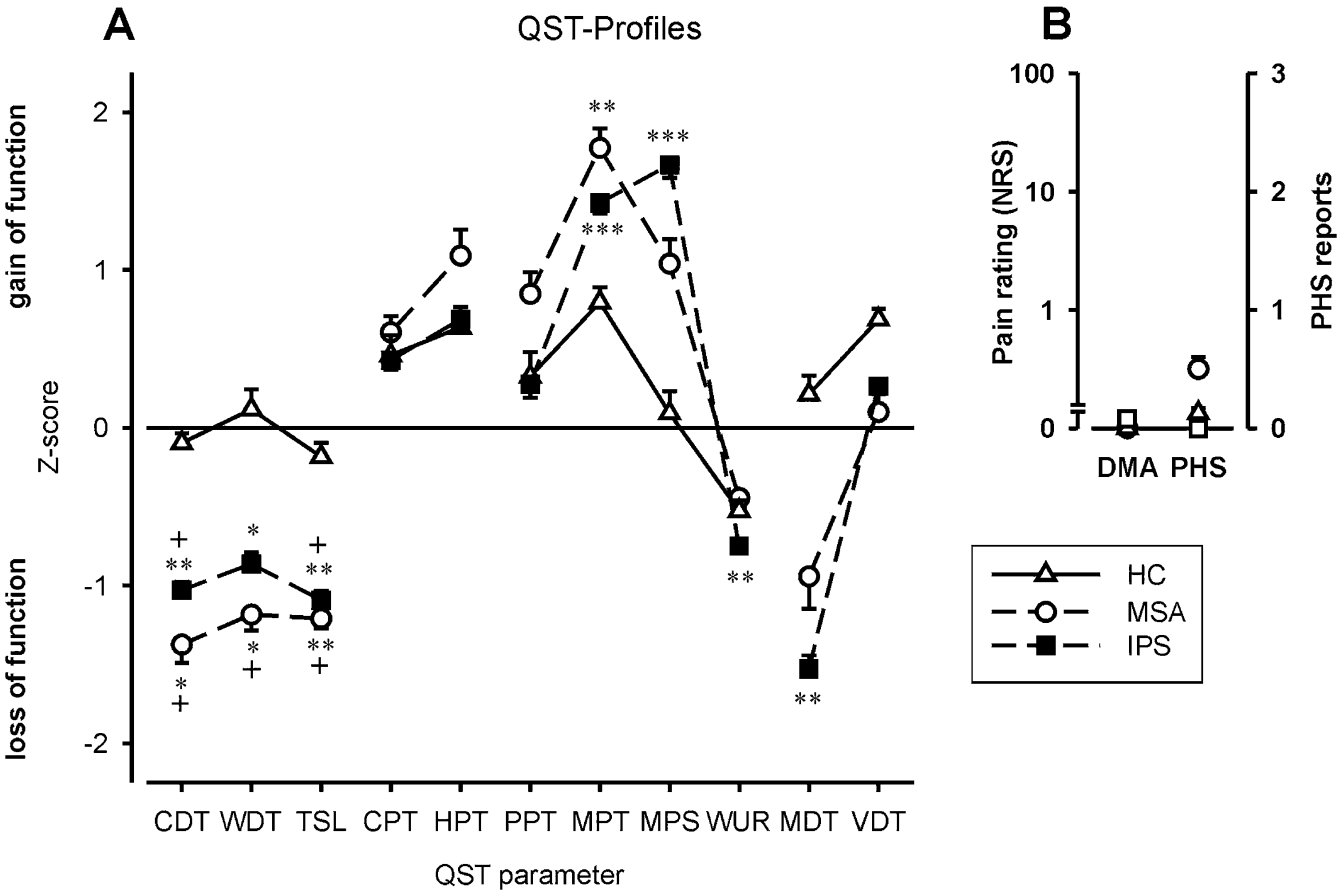
Presents somatosensory profiles of healthy controls (open triangles), PD patients (filled squares), and MSA patients (open circles). *CDT* cold detection threshold, *WDT* warm detection threshold, *TSL* thermal sensory limen, *CPT* cold pain threshold, *HPT* heat pain threshold, *PPT* pressure pain threshold, *MPT* mechanical pain threshold, *MPS* mechanical pain sensitivity, *WUR* wind-up ratio, *MDT* mechanical detection threshold, *VDT* vibration detection threshold, *DMA* dynamic mechanical allodynia, and *PHS* paradoxical heat sensations. *p < 0.05; **p < 0.01; ***p < 0.001: level of significance compared to the normative data of the DFNS (*z* score 0; SD 1). ^**+**^*p* < 0.05: level of significance between MSA vs. HC and PD vs. HC, respectively

QST parameters of healthy controls (HC) were not different from the normative data set of the DFNS (for details, see Fig. 3).

PD and MSA presented with impaired cold (CDT-z-scores: PD − 1.03 ± 0.06, *p* < 0.01; MSA − 1.38 ± 0.12; *p* < 0.05) and warm detection thresholds (WDT-z-scores: PD − 0.86 ± 0.08; MSA − 1.19 ± 0.1; both *p* < 0.05). Consecutively, the thermal sensory limen (TSL) as a compound measure was also impaired (z-scores: PD − 1.09 ± 0.06; MSA − 1.21 ± 0.06, both *p* < 0.01).

The mechanical detection threshold was higher in PD only (MDT-z-score − 1.53 ± 0.9, *p* < 0.01), indicating loss of function (tactile hypoesthesia). However, decreased z-scores for mechanical pain thresholds were seen in both groups (PD, MSA; z-score: PD 1.43 ± 0.07; MSA 1.77 ± 0.12; both *p* < 0.001) as a sign of mechanical hyperalgesia. Additionally, mechanical pain sensitivity (MPS) was significantly increased in PD (z-score: 1.67 ± 0.08; *p* < 0.001) but not in MSA (z-score 1.04 ± 0.15, n.s.; for details, see Fig. 3).

Further signs of central (WUR and DMA) or peripheral sensitization (PPT) were not found in MSA and PD.

#### Comparison between HC, PD, and MSA

Thermal detection thresholds were different between the three groups [*F*(4.66) = 2.55, *p* < 0.048]. Post hoc analysis revealed that cold detection (CDT) was impaired in PD and MSA compared to HC (both *p* < 0.05). Moreover, we found elevated warm detection thresholds (WDT) in MSA compared to HC (*p* < 0.05); in PD, there was a non-significant trend (*p* = 0.07). Consecutively, thermal sensory limen [TSL; *F*(2.34) = 3.298; *p* = 0.049] was different in PD and MSA compared to HC (both *p* < 0.05). Please see Fig. 3 for details.

Thermal pain thresholds (CPT and HPT), mechanical detection thresholds (MDT and VDT), and mechanical pain thresholds (MPT, PPT, and ALL) were not different between groups. The same holds true for the response to suprathreshold stimuli (MPS and WUR). Paradoxical heat sensations (PHS) were reported by one HC, five patients with PD, and three patients with MSA (Fisher’s exact test, n.s.). For details, see Fig. 3.

## Discussion

The present study provides evidence of autonomic and somatosensory dysfunction in PD and MSA. In both patient groups, we found evidence for sympathetic impairment. This impairment seems to be of peripheral origin in PD, but not in MSA. Moreover, we found evidence for small fiber neuropathy in both patient groups, including mechanical hyperalgesia. These findings support pathophysiological concepts of MSA and PD.

### The investigation toolbox

OH can occur in patients with central or peripheral autonomic nervous system disorders (neurogenic orthostatic hypotension), adrenal insufficiency [25], or hypovolemia (non-neurogenic orthostatic hypotension) [29]. OH testing assesses noradrenergic and adrenergic function. Forehead cooling activates facial receptors that relay to the trigeminal sensory nucleus and via interneurons to the vasomotor and cardiac centers in the medulla [8]. It induces peripheral reflex vasoconstriction, which is a function of peripheral NE release [35]. The cutaneous application of NE via microdialysis exclusively assesses the sensitivity of peripheral NE receptors on cutaneous arterioles. With high concentrations of NE, the system is saturated and vasoconstriction is maximal; with physiological NE concentrations, a sensitivity change of NE receptors on arterioles can be assessed. Cannon’s law of denervation describes a supersensitivity of receptors if peripheral sympathetic neurons degenerate [6].

Quantitative sensory testing (QST; DFNS protocol) is an established and reliable method to detect sensory abnormalities over all nerve fiber classes, including central pathways [20, 43]. QST assesses sensory loss, but in contrast to, e.g., nerve conduction, it could also assess gain of function signs like hyperalgesia as a result of peripheral and central nociceptive sensitization [33].

### The autonomic failure

In MSA, autonomic failure is part of the diagnosis, and its severity is normally more devastating compared to PD [31]. We found significant orthostatic hypotension and a decreased peripheral vasoconstriction after cold stress in MSA. NE microdialysis, however, was closer to HC than to PD. These findings support that degeneration of central autonomic neurons is the main reason for autonomic failure in MSA [16], whereas peripheral noradrenergic innervation might be relatively preserved. Accordingly, in MSA, α-synuclein deposits are present only in a minority of the noradrenergic fibers in the skin [14]. Furthermore, a few MSA patients have cardiac sympathetic denervation indicated by reduced cardiac uptake of MIBG [37]. Thus, the peripheral autonomic nervous system remains relatively preserved in MSA.

Autonomic failure in PD is different [40, 52, 53]. Our results indicate orthostatic hypotension, preserved cold stress-induced vasoconstriction but stronger vasoconstriction after microdialysis of physiological NE concentrations. The latter provided evidence for denervation hypersensitivity of NE receptors on cutaneous blood vessels [6]. Previous histological studies have visualized moderate degeneration of peripheral sympathetic fibers in PD [9]. Skin samples suggested that phosphorylated α-synuclein (P-α-synuclein) deposits led to the degeneration of the peripheral autonomic nerve fibers [14]. Thus, our results suggest that PD autonomic failure is, at least partially, due to peripheral noradrenergic failure.

### Somatosensory function

Sensory function in treated PD was reported to be heterogeneous [54] with different patterns of sensory loss (hypesthesia) and sensory gain (hyperalgesia) [36, 56]. In the present study, we applied the widely accepted QST study protocol of the DFNS [43]. In PD and MSA, we found increased thermal detection thresholds, indicating Aδ and C fiber dysfunction or degeneration. This pattern of sensory abnormalities resembles the pattern found in small fiber neuropathies [11, 21]. Indeed, in MSA and PD, α-synuclein deposits were described in epidermal and subepidermal nerve fibers [14]. However, none of the PD or MSA patients reported sensory symptoms or any clinical signs of small fiber neuropathies, such as neuropathic pain [11]. Additionally, the mechanical detection threshold was increased in PD only, correlating to large fiber neuropathy, which has been described before in PD [10]. The mechanism why neuropathy develops in neurodegenerative extrapyramidal diseases is unclear. Impairment of vitamin B12 and folate uptake might be one reason [7], or peripheral somatosensory nerve degeneration is an inherent part of PD and MSA pathology.

Another remarkable QST finding was hyperalgesia for pinprick stimuli, which was found in PD and MSA. Pinprick hyperalgesia indicates central (i.e., spinal) sensitization [2]. Previous studies have been inconsistent. While in early PD pain processing was found to be unaltered [18], a recent meta-analysis [47] and a pain–evoked potentials study [55] revealed hyperalgesia to painful stimuli as common in PD. One explanation for hyperalgesia is the lack of dopamine which physiologically mediates descending pain inhibition to the spinal cord [34]. Conversely, oral dopamine increases pain thresholds [5]. In addition, sensory–motor integration in the cortex of PD patients is abnormal, particularly in PD patients with pain. This abnormal integration was independent from L-dopa administration [48]. Thus, PD-related disturbances of spinal or cortical pain processing might be responsible for mechanical hyperalgesia in our patients. Peripheral hyperalgesia in subclinical neuropathy without pain symptoms is less likely but not excluded.

### Limitations

The number of participants differs between the groups. However, as PD occurs more frequently than MSA, our results are justified. Moreover, we compared the QST results from our patients not only to our control group but also to the DFNS normative data set. We did not assess clinical autonomic symptoms, which were present in MSA by definition, but might be present probably also in PD where they might precede motor symptoms [41]. We did not use the MDS-URPDS-III but the former URPDS-III. We investigated early-to-intermediate PD patients which can be deferred from the low L-dopa equivalent daily doses and the low score in the URPDS. Dyskinesia, a symptom that is not included in the URPDS-III but in the MDS-URPDS-III was not prevalent in our patients’ group.

### Conclusion

MSA and PD look similar in peripheral sympathetic and somatosensory dysfunction. They differ, however, substantially in the mechanisms behind these disturbances. The results of our study may help to find a targeted treatment of autonomic and pain symptoms in both diseases.

## Data Availability

The data are available from the corresponding author upon reasonable request.
